# Monosodium Glutamate Treatment Elevates the Immunoreactivity of GFAP and S100β in Caudate Nucleus of the Striatum in Rats

**DOI:** 10.3390/biomedicines12122763

**Published:** 2024-12-04

**Authors:** Karol Rycerz, Aleksandra Krawczyk, Jadwiga Jaworska-Adamu, Marcin B. Arciszewski

**Affiliations:** Department of Animal Anatomy and Histology, Faculty of Veterinary Medicine, University of Life Sciences, Akademicka 12, 20-033 Lublin, Poland; karol.rycerz@up.lublin.pl (K.R.); jadwiga.jaworska@up.lublin.pl (J.J.-A.); mb.arciszewski@wp.pl (M.B.A.)

**Keywords:** monosodium glutamate, brain, astrocytes, GFAP, S100β, excitotoxicity, caudate nucleus

## Abstract

Background Monosodium glutamate (MSG) in its anionic form, glutamate, is one of the main excitatory amino acids. Excess of this neurotransmitter may lead to excitotoxicity affecting neurons and astrocytes responsible for glutamate metabolism in different brain areas of animals. The aim of the study was to investigate the immunoreactivity of glial fibrillary acidic protein (GFAP) and S100β protein in the caudate nucleus of rats under the condition of elevated glutamate levels. Methods: Fifteen rats were divided into a control group receiving saline and MSG2 and MSG4 groups receiving 2 g/kg b.w. MSG and 4 g/kg b.w. MSG, respectively, for 3 days. An immunohistochemical reaction was conducted on frontal sections containing the caudate nucleus with use of antibodies against GFAP and S100β. Results: Analyses indicated elevated density of astrocytes immunoreactive for the studied proteins in the caudate nucleus in animals receiving MSG. The studied glial cells also demonstrated increased immunostaining intensity for both GFAP and S100β immunoreactive cells especially in the MSG4 group. The number of GFAP-positive processes in astrocytes was similar in all studied groups. Conclusions: The studies demonstrate a potential response of astrocytes to the effect of MSG administration in the caudate nucleus. It was shown that GFAP- and S100β-positive astrocytes in the caudate nucleus may act differently, suggesting distinct roles of these proteins against glutamate excitotoxicity.

## 1. Introduction

The anionic form of monosodium glutamate (MSG) is glutamic acid, glutamate (Glu), which belongs to the biogenic amino acids [1]. It is one of the main excitatory amino acids in the central nervous system (CNS) which is present in about 80–90% of synapses [2,3]. Excess of Glu may lead to excitotoxicity causing cellular lesions and even death of neurons and glia [4]. Stimulation of metabotropic Glu receptors (mGluR1 and mGluR5) causes the opening of ion channels and the influx of Ca^2+^ into neurons and glial cells [2,3,4]. Glial glutamate transporters glutamate/aspartate transporter/excitatory amino acid transporter 1 (GLAST/EAAT1) and glutamate transporter-1/excitatory amino acid transporter 2 (GLT-1/EAAT2) play an important role in MSG metabolism. These transporters capture excess Glu from the synaptic clefts. Then, astrocytes degrade Glu to non-excitatory glutamine which is transferred to neurons for resynthesis [5]. However, in pathological conditions, Glu neurotransmitter is released in excess into the intercellular space. Released in high concentrations, it acts on N-methyl-D-aspartate (NMDA) receptors found in both neurons and glial cells, causing hyperexcitability of neurons, disrupting their functions and leading to cellular death [4,5,6].

This phenomenon may affect the astrocytic distribution and proportions of glial fibrillary acidic protein (GFAP) and S100β protein in cells. GFAP is a protein that is a part of the glial intermediate filaments that constitute the cytoskeleton of glial cells. It affects the stability, mobility and mitotic activity of astrocytes [7]. S100β is a calcium-binding protein that localizes in the nucleus and cytoplasm of stellate glial cells. It regulates many intra- and extracellular processes such as the phosphorylation of crucial synaptic proteins, maintenance of homeostasis in the CNS and regulation of proliferation, transcription, differentiation, enzymatic activity and metabolism [8]. It regulates the functioning of astrocytes, microglia and neurons [9,10]. MSG causes astrogliosis, increasing the immunoreactivity of GFAP-positive astrocytes as a response to the toxic effect of Glu in different areas of the brain [11,12,13]. Glu increases the extracellular Ca^2+^ levels, which also contributes to the activation of astrocytes and increased reactivity of S100β protein in relation to its buffering functions [14,15]. Therefore, extrasynaptic levels of Glu are regulated by astrocytes, which in the case of excess levels of Glu may change their functions to buffer and take Glu up from extracellular matrix [11,12,13,14,15,16]. This was already demonstrated in striatum [16].

Previous studies have revealed the neurotoxic effect of MSG in many areas of the animal brain. Oral administration of this substance to mice at doses of 40 and 80 mg/kg b.w. for 28 days caused changes in neurons and the appearance of reactive astrocytes as evidenced by an increase in the density of these glial cells and an increase in the thickness of their processes in the cortex, hippocampus and cerebellum [17]. Moreover, MSG caused degenerative lesions in hippocampal neurons in rats receiving this substance both per os and subcutaneously for 10 days [18]. Other authors have shown a similar effect of MSG which was administered to rats in food for 14 days in the cerebellum, where there was an increased cellular disruption and the presence of granule and Purkinje cell degeneration [19,20]. When considering MSG dosing, it should be noted that 2 g/kg b.w. of MSG induced excitotoxic neuronal damage, increased levels of free oxygen radicals and increased levels of beta amyloid in the hippocampus [18,21]. MSG administered at a dose of 4 g/kg b.w. induced cell death with cytoarchitectonic lesions in the hippocampus associated with hyperexcitability and changes in motor behavior [21].

Our previous studies have also shown the effect of MSG on both neurons and glial cells in the hippocampus, periaqueductal gray matter and dorsal raphe nucleus [9,22,23,24].

So far, the effect of MSG on immunoreactivity for GFAP and S100β in astrocytes of the caudate nucleus of the striatum has not been studied. Striatum is responsible for, among others, procedural memory, reinforcement of instrumental behaviors, regulation of movements and cognitive functions. The caudate nucleus as a part of the dorsal striatum is responsible for motor learning, consolidation of motor skills and performance of habitual activities and is associated with addictions and drug-seeking behavior [5,25,26,27]. It was shown that this nucleus is involved in the pathology of such conditions as Huntington’s disease, in which the number of GFAP- and S100β-positive cells is increased, or a small ischemic lacunar stroke in the territory of a single deep perforating lenticulostriatal artery corresponding to basal ganglia [28,29]. Glial cells from the caudate nucleus may undergo carcinogenesis and be the source of gliomas [30,31].

The caudate nucleus contains mainly GABA-ergic neurons [32]. Other neuronal cell types are acetylcholinergic, serotonergic and dopaminergic. Recent studies have shown that glutamate from the cerebral cortex and thalamus is essential for the release of acetylcholine in the striatum, particularly in decision-making reward trials [33]. Moreover, it has already been proven that Glu inhibits the release of dopamine (DA) in the striatum, which occurs via activation of group I metabotropic receptors. The source of dopamine in this area is the nigrostriatal pathway in which long-range axons originating from the substantia nigra of the midbrain and the ventral tegmental area release DA [34,35]. It was found that there is a dynamic relationship between dopamine and acetylcholine during decision making and regulation of cholinergic interneurons in the striatum [33,34,35,36]. Based on these data, we hypothesized that glutamate may affect not only striatal neurons but also glial cells in this area. Hence, the aim of the study was to perform immunohistochemical assessment of the reactivity of GFAP and S100β proteins used as markers of astrocytes in the CNS after subcutaneous administration of MSG to rats. GFAP-immunoreactive (GFAP-IR) and S100β-immunoreactive (S100β-IR) astrocytes in the striatum constitute two distinct populations of stellate glial cells and their response to harmful factors may differ [28].

## 2. Materials and Methods

### 2.1. Animals, the Study Model and Sample Collection

The studies with the use of animals were conducted with the consent of the Second Local Ethical Committee in Lublin (Agreement no. 7/2011). Fifteen Wistar rats (body weight 220–250 g) were used in the experimental model. The rats were kept in cages in constant conditions, at a temperature of 20–22 °C, 60% air humidity and a 12 h light/12 h dark cycle. The animals had constant access to standard rat chow and water. The 60-day-old rats were divided into 3 groups of 5 individuals in each group. The control group (C) received subcutaneous injections of saline for 3 consecutive days. At the same time, two other groups received subcutaneous injections of MSG (Sigma-Aldrich, St. Louis, MO, USA, 49621) prepared in physiological saline in doses of 2 g/kg b.w. (MSG2 group, *n* = 5) and 4 g/kg b.w. (MSG4 group, *n* = 5). The dose was selected based on literature data [21].

Twenty-four hours after the last injection, the animals were euthanized. Brains were collected, fixed in 10% formalin, dehydrated with a graded scale of increasing concentrations of alcohols, cleared in xylene and embedded in paraffin blocks by routine histological techniques. The blocks were then cut with a microtome (Microm HM 325, Thermo Scientific, Walldorf, Germany) into 4 µm thick frontal sections containing the caudate nucleus (from A5340 µm to A4620 µm according to a stereotaxic atlas [37]), and every fifth section was mounted on SuperFrost Plus (Gerhard Menzel B.V. and Co. KG, Braunschweig, Germany) slides.

### 2.2. Immunohistochemical Staining

The obtained sections were deparaffinized in xylene and rehydrated in alcohol. All antibodies and reagents were purchased from Sigma Aldrich (St. Louis, MO, USA) and diluted in 0.5 M Tris (Tris-buffered saline T5912, Sigma Aldrich, St. Louis, MO, USA) according to the manufacturer’s recommendations. Then, the sections were incubated in 0.4% H_2_O_2_ at room temperature for 30 min to inhibit endogenous peroxidase activity. In the next step, a normal goat serum (G9023) was used at room temperature for 20 min to prevent non-specific background staining. Antigens were retrieved by heating in the microwave three times for 5 min with citrate buffer (pH = 6.0). Then, the sections were incubated with primary antibodies at 4 °C for 16 h. Half of the sections were treated with a polyclonal anti-GFAP antibody produced in rabbits (G9269, 1:80) and half with a monoclonal anti-S100β antibody (S2532, 1:1000) produced in mice. The sections were then treated with the species-specific secondary polyclonal anti-rabbit IgG antibody (A9169, 1:400) and polyclonal anti-mouse IgG (A9917, 1:200) at room temperature for 1 h. 3,3′diaminobenzidine tetrachloride (DAB, 32750) was used as a chromogen to stain the antigen–antibody complexes. The sections were then washed in distilled water and counterstained with Mayer’s hematoxylin. The sections were subsequently dehydrated, cleared in xylene and mounted in DPX (Fluka, Buchs, Switzerland). The stained sections were photographed and assessed qualitatively and quantitatively under the Olympus BX51 microscope (Olympus, Tokyo, Japan) with a digital camera (Olympus Color View III). The assessment of the specific binding of the antibodies used was performed using a control procedure. Positive specificity control was conducted in the hippocampus and compared with our previous study [9]. Sections not exposed to primary antibodies acted as a negative control for the specificity of the reaction which was performed by omitting the primary antibody and replacing it with normal goat serum. Control sections showed no affirmative immunohistochemical reaction.

### 2.3. Quantitative Analyses

Twenty sections immunostained for GFAP and S100β from each animal were randomly selected for the microscopic assessment. Two micrographs were taken from each section, archived and further analyzed in the Cell^D program 2.8 (build 1233) (Olympus, Tokyo, Japan). A grid of squares of 0.03 mm^2^ was imposed on randomly selected microphotographs showing the caudate nucleus. GFAP-IR cells and S100β-IR cells were counted in the squares from different images until reaching the result from 100 grid squares. In the micrographs with the reaction for GFAP, the number of the GFAP-IR cell processes was also counted. The number of the processes was counted in 100 random astrocytes. The assessment of the reaction intensity was also conducted and was based on methods presented in the literature [38,39]. The cells with visible brown reaction product were selected to measure the intensity of staining in a region of interest of 1 mm^2^. The highest color value is 255 and represents a white color. The darkest, black color is represented by the value of 0. Hence, the results were inverted and presented as optical density (OD) which shows higher values for darker staining color. Additionally, the intensity of free space was measured to standardize the differences in light exposure. Hence, OD was calculated as: OD = 255 − (255x/y) where x is the measured intensity in the cell and y is the measured intensity of free space [39]. To classify the intensity levels of staining, thresholds were used, where strong reaction intensity was represented by results above OD = 195, medium reaction intensity by OD = 135–195 and weak reaction intensity as OD = 75-194. OD results below 74 were considered as negative.

### 2.4. Statistical Analyses

The results were presented as mean ± standard deviation. The Shapiro–Wilk test was performed to assess the normal distribution of the data and Levene’s test to assess the homogeneity of variance. The obtained results indicated the need to use non-parametric tests for the comparison of the results. The Kruskal–Wallis test was used to compare means between every individual group. The significance factor was set at α = 0.05.

## 3. Results

### 3.1. GFAP Immunoreactivity Results in the Studied Animals

**Figure 1:**
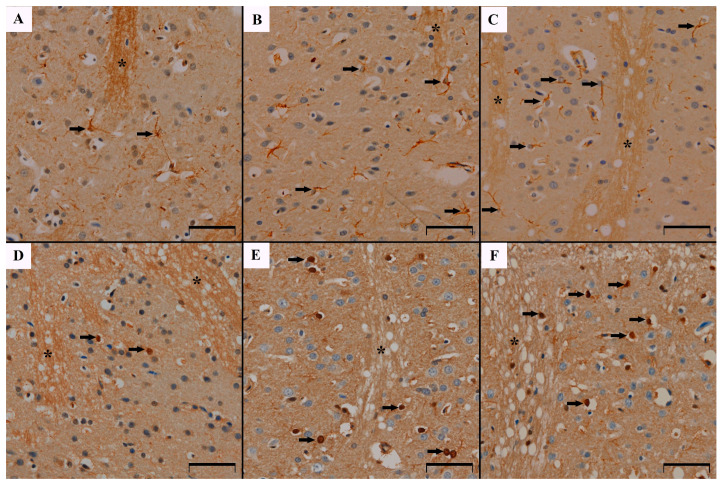
Immunohistochemical staining of caudate nucleus area using anti-GFAP antibody in Control group (**A**), MSG2 group (**B**), MSG4 group (**C**) and anti-S100β antibody in Control group (**D**), MSG2 group (**E**) and MSG4 group (**F**). An increased number of GFAP-IR and S100β-IR astrocytes in MSG2 and MSG4 groups is demonstrated. GFAP-IR astrocytes contain short and few processes. The arrows indicate the astrocytes. The asterisks indicate the neuronal fibers within the caudate nucleus. Scale bar 50 µm.

**Figure 2:**
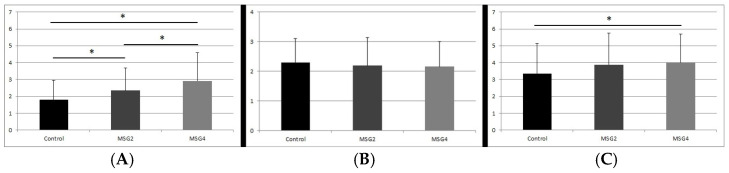
Average density of GFAP-IR astrocytes (**A**), average number of GFAP-IR astrocytic processes (**B**) and average density of S100β-IR astrocytes (**C**) in caudate nucleus of the studied groups of rats (Control, MSG2 and MSG4). Data show mean values with standard deviation. * Statistically significant differences between the studied groups, Kruskal–Wallis, *p* < 0.05.

Immunohistochemical studies showed the presence of the GFAP protein reaction product in the perikarya and astrocyte processes in the caudate nucleus in all groups of the studied animals. The cells were loosely scattered between the bundles of nerve fibers (Figure 1A–C). It was shown that in rats receiving MSG at doses of 2 g/kg b.w. and 4 g/kg b.w. the density of astrocytes was statistically significantly higher than in the group of control animals (1.8 ± 1.2 control group vs. 2.34 ± 1.3 and 2.92 ± 1.7 respectively, *p* < 0.05 Kruskal–Wallis). This shows that the cell density in the MSG4 group is 39% higher than in the control group. The cell density in the MSG2 group is 19% higher than in the control group. A statistically significant difference in astrocyte density was also observed between the MSG2 and MSG4 groups, suggesting that cell density increased (approximately 20% difference) depending on the dose (*p* < 0.05, Kruskal–Wallis) (Figure 2A). Moreover, a decreasing trend was demonstrated in the number of protrusions, however, these differences were not statistically significant (*p* > 0.05, Kruskal–Wallis) (Figure 2B).

### 3.2. Results of GFAP Reaction Intensity

**Figure 3:**
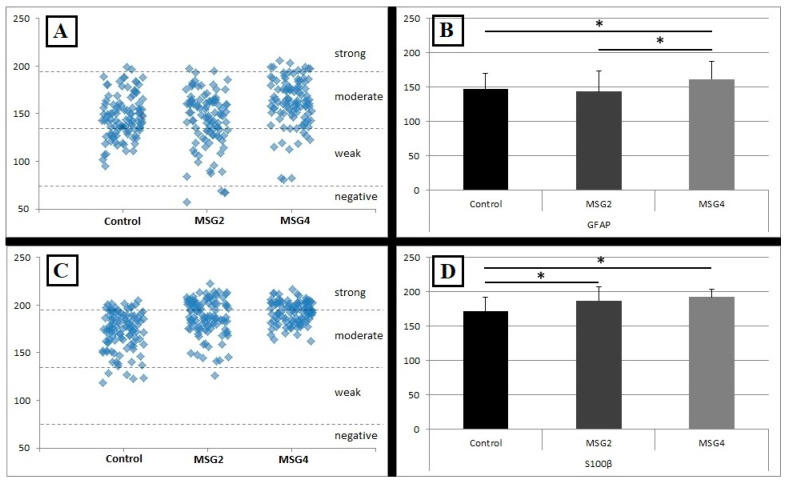
Optical density of GFAP-IR astrocytes (**A**) and S100β-IR astrocytes (**C**) in 100 cells divided between strong, moderate, weak and negative reaction intensity. An average optical density of GFAP-IR astrocytes (**B**) and average optical density of S100β-IR astrocytes (**D**) in the studied groups of rats (Control, MSG2, MSG4). Data in B and D show mean values with standard deviation. * Statistically significant differences between the studied groups, Kruskal–Wallis, *p* < 0.05.

Most GFAP-IR astrocytes were characterized by a moderate reaction intensity. In the control group, it was as much as 69% of the examined cells, similarly to the MSG2 group, where the percentage of moderately stained cells was 65%. More moderately stained cells, i.e., 80%, were found in the MSG4 group of rats. In this group, there were also 8% strongly stained cells. In the MSG2 group, the presence of 4% of cells assessed as negative was also demonstrated (Figure 3A).

These results are confirmed by the assessment of the average reaction intensity as OD in astrocytes positive for GFAP which was statistically significantly different between individual groups of animals. An upward trend in changes depending on the MSG dose was demonstrated. The highest mean intensity of the reaction was observed in astrocytes in animals receiving the higher dose of MSG and it was statistically significantly different from the intensity of GFAP-positive cells from the control group (increased by approximately 6%, *p* < 0.05 Kruskal–Wallis) and the MSG2 group (increased by approximately 8%, *p* < 0.05, Kruskal–Wallis). However, there was no statistically significant difference between the control group and the MSG2 group (Figure 3B).

### 3.3. S100β Immunoreactivity Results in the Studied Animals

S100β-positive astrocytes were present in the caudate nucleus in both the control group and the groups of animals receiving MSG. They were loosely scattered in this area between the bundles of nerve fibers. The reaction product was located mainly in the cell nuclei of the studied glia (Figure 1D–F). The density of S100β-positive cells in the caudate nucleus was higher in both groups of animals receiving MSG compared to the control group (3.35 ± 1.8 control group vs. 3.88 ± 1.9 MSG2 group and 4.01 ± 1.7), however, a statistically significant difference occurred only between the control group and MSG4 (increased by approximately 6%, *p* < 0.05, Kruskal–Wallis) (Figure 2C).

### 3.4. Results of S100β Reaction Intensity

Results from immunostaining reaction intensity of S100β-IR astrocytes demonstrated a clear upward trend which was noticeable between the individual groups. In the control group, the majority of cells, i.e., as much as 83%, showed a moderate level of immunostaining intensity, and cells with strong staining constituted 12%. In the MSG2 and MSG4 groups, almost half of the cells showed a moderate level of immunostaining (56% and 51%, respectively) and a large part of the cells showed strong staining intensity (43% and 49%, respectively) (Figure 3C).

Comparison of the average immunostaining intensities between the groups showed that in the caudate nucleus of animals receiving MSG at both lower and higher doses, astrocytes are more intensively immunostained than in the control group (*p* < 0.05 MSG2 and *p* < 0.05 MSG4), showing an increase in the intensity by about 6% and 8%, respectively. Differences in mean intensity were independent of dose (MSG2 vs. MSG4, Kruskal–Wallis *p* > 0.05) (Figure 3D).

## 4. Discussion

The results of this study indicated an elevated density of caudate nucleus astrocytes immunoreactive for GFAP in both groups of rats receiving MSG and for S100β protein in rats receiving a higher dose of MSG. The studied glial cells also demonstrated increased immunostaining intensity for both GFAP and S100β immunoreactive cells especially in the group of rats receiving a higher dose of MSG. The number of GFAP-positive processes in astrocytes was similar in all studied groups.

Astrocytes in the striatum play a crucial role in the modulation of neurotransmission, forming a network of numerous connections. They participate in synaptic communication and synaptic plasticity [5]. Brain homeostasis is affected by the excess of Glu through the effect on astrocytic NMDA receptors. Activation of these receptors in astrocytes causes neuroprotection by a mechanism which leads to the release of calcium ions from the endoplasmic reticulum and the phosphorylation of the p35 protein which leads to the synthesis and release of glutathione with antioxidant activity [40]. On the other hand, long-term activation of these receptors can also lead to changes in the metabolism of astrocytes and the loss of proteins such as glutathione synthetase or water channel protein aquaporin-4 [40]. In the striatum, the excess of Glu inhibits the release of DA through the activation of mGluR1 receptors via the CA^2+^-dependent pathway. The regulation of extrasynaptic Glu levels in the striatum is mainly mediated by astrocytes using GLT-1/EAAT2 transporters [16]. Changes in the extrasynaptic Glu levels in the striatum and DA levels from the nigrostriatal pathway may induce lesions in astrocyte function and structure [41].

Our findings show that the density of GFAP-IR astrocytes significantly increased in the examined surface area in the caudate nucleus of both groups of animals receiving a lower and higher dose of MSG. These changes were dose-dependent. Our previous studies also demonstrated an increase in GFAP-IR astrocyte density in the stratum lacunosum moleculare layer of the hippocampus of 10-day-old rats receiving MSG [9].

The results of our studies are in line with the studies of other authors who investigated other brain areas. It has been shown that MSG increases the number of GFAP-positive astrocytes and the average area percentage of immunoreactive GFAP, indicating active astrogliosis as a secondary response to toxic insult of Glu in the hippocampus, dentate gyrus and cerebellar cortex [11,12,13]. GFAP concentration correlates with the severity of injury, which was confirmed by computed tomography and magnetic resonance imaging studies [42]. In our studies, we also demonstrated an increase in the moderate intensity of GFAP-IR immunostaining of astrocytes in the caudate nucleus in animals receiving a higher dose of MSG. In this group, MSG4, as many as 80% of glial cells showed moderate immunostaining intensity, while in the control group and MSG2, moderate immunostaining intensity was shown by 69% and 65% of cells, respectively, with a higher percentage of weakly stained cells and those which were considered as negative. Similar results were obtained by other authors, who described the GFAP-IR reactivity of astrocytes in the hippocampus after MSG as strong [11,12]. Immunohistochemical studies of astrocytes in the hippocampus showed massive immunoreactivity of GFAP in MSG-treated rats, which was associated with the effect of neuroexcitation [13]. Using the ELISA method, a fivefold increase in GFAP was demonstrated in the brain of Sprague Dawley rats after the administration of 4 mg/kg b.w. MSG by oral gavage [43]. Our studies showed an increase in both astrocyte density and the intensity of the GFAP reaction in the caudate nucleus of rats after MSG. This may be related to the excitotoxic effect of MSG causing an increase in the concentration of extracellular Glu and the stimulation of metabotropic mGluR1 and mGluR5 receptors [4,6]. In this way, calcium ions are released, leading to excessive mitochondrial activity in cells, which results in damage to the cytoskeleton, cell membrane and DNA mainly in nervous cells [4,6,44]. Consequently, there is an increased reactivity of astrocytes for neuroprotection and uptake of Glu excess [11,42,45]. Another mechanism that may explain the increase in the density and immunostaining intensity of GFAP-IR astrocytes may be related to damage to DA neurons and disintegration of neurotransmitter flow in the nigrostriatal pathway under the influence of Glu. Free radicals generated as a result of Glu excitotoxicity may damage dopaminergic neurons, which are particularly more sensitive to Glu neurotoxicity than other neurons [46,47]. As a result of damage to DA neurons in the substantia nigra, there is a local increase in GFAP levels in the striatum, which is associated with elevated Glu levels. It has been shown that this increase in GFAP immunoreactivity can be up to 4-fold and is associated with an increase in the reactivity and number of astrocytes in the striatum [41,48]. Astrocytes modulate neurotransmission in the striatum, e.g., by influencing dopaminergic transmission, which in turn affects the level of glutamine, which is a substrate for the production of Glu [5].

In contrast to our studies, where it was shown that there is no statistically significant change in the length of astrocyte processes in the striatum, other authors indicated increased immunoreactivity of processes in the hippocampus of rats [12,13]. This may be related to the reactivity of astrocytes other than GFAP-IR in the striatum than in the hippocampus. It is known that not all astrocytes in the striatum are GFAP reactive and there are other fractions of these cells. It has been shown that GFAP-IR and S100β-IR astrocytes represent two separate populations of astrocytes in the striatum and their response may be different in different disease conditions in terms of astrogliosis or distribution [28].

Our studies also showed that in animals receiving Glu there was an increase in the density of S100β-positive cells in the caudate nucleus of the striatum. However, this increase was statistically significant only in the MSG4 group of animals. Nevertheless, the intensity of immunostaining of S100β-IR astrocytes was clearly higher in both MSG2 and MSG4 groups. In both MSG2 and MSG4 groups, 43% and 49% of cells, respectively, showed a strong reaction intensity compared to S100β-positive astrocytes in the control group, where strongly immunostained cells constituted only 12% of astrocytes. It has been shown that in neurodegenerative diseases and other conditions leading to neuronal cell death, the level of S100β protein increases in astrocytes, extracellular space and in serum. Some authors consider this protein to be a marker of the severity of some neurodegenerative diseases [14,28,42,45]. It is known that one of the mechanisms of Glu-dependent excitotoxicity is excessive CA^2+^ release [15]. S100β is a calcium-binding protein, hence in a condition of unbalanced levels of intracellular CA^2+^ the S100β immunoexpression may increase to buffer the excess of these ions [14]. In addition, S100β causes an increase in the frequency of mitotic divisions, which affects the cell density in the different areas of the CNS [9]. Moreover, S100β in the extracellular space modulates the uptake of Glu by astrocytes, leading to the activation of these cells [45]. In this way, astrocytes play a neuroprotective role in the CNS against Glu excitotoxicity and this is closely related to the appropriate nanomolar concentration of S100β. Glu induces the expression of S100β receptors called receptors for advanced glycation end products (RAGEs) in neurons, which leads to the activation of nuclear factor kappa β, inhibiting neuronal death [15,49]. Moreover, similarly to the increase in the density and immunoreactivity of the GFAP protein, the S100β protein is also involved in the striatum in the astrocyte response associated with DA-ergic neurodegeneration. It was found that the loss of DA neurons was followed by an increase in the level of S100β in the striatum [48]. Hence, the increase in the density and immunostaining intensity of S100β-IR astrocytes in our study may be, on the one hand, a response to the decrease in dopaminergic transmission in the striatum and, on the other hand, a protective effect of this protein against Glu excitotoxicity by buffering CA^2+^ excess.

The study has a limitation that needs to be acknowledged. The MSG is used as a flavor enhancer in food, thus administrating MSG orally should be considered as it was suggested in other studies to take into account the influence of microflora and metabolic changes that appear in the intestines [17,18,19,20]. However, to avoid discrepancies related to the calculation of the total dose of MSG, we applied a subcutaneous administration of MSG, which was also related to the methodology presented in some other studies [21]. We believe that this limitation does not undermine the novelty of our study.

## 5. Conclusions

In conclusion, the study showed a significant increase in the density and immunostaining intensity of GFAP-IR and S100β-IR astrocytes in the caudate nucleus of the striatum in rats receiving MSG. Studies suggest a potential response of astrocytes to changes induced by excess of Glu in order to maintain the neuronal microenvironment and proper functioning of the striatum, which may involve different mechanisms related to excitotoxicity. Hence, this experiment provides important information on how excess of Glu may affect critical aspects of CNS functioning. The observed changes were dose-dependent in GFAP-IR astrocytes and independent in S100β-IR astrocytes, suggesting differential reactivity of GFAP and S100β-IR astrocytes to Glu’s effect in the caudate nucleus. Further studies concerning other hallmark proteins for astrocytes like vimentin in the context of cell activation and development, or glutamate transporter 1 responsible for glutamate uptake from synaptic clefts, should be performed to clarify different astrocyte signaling pathways under the influence of MSG in the caudate nucleus.

## Data Availability

Data are contained within the article.
